# Draft Genome Sequence of the Formaldehyde-Resistant Fungus *Byssochlamys spectabilis* No. 5 (Anamorph *Paecilomyces variotii* No. 5) (NBRC109023)

**DOI:** 10.1128/genomeA.01162-13

**Published:** 2014-01-09

**Authors:** Takuji Oka, Keisuke Ekino, Kohsai Fukuda, Yoshiyuki Nomura

**Affiliations:** Department of Applied Microbial Technology, Faculty of Biotechnology and Life Science, Sojo University, Kumamoto City, Japan

## Abstract

*Byssochlamys spectabilis* no. 5 (anamorph *Paecilomyces variotii* no. 5) (NBRC109023) was isolated from a soil sample in 2001 in Kumamoto Prefecture, Japan. This fungus is highly resistant to formaldehyde. Here, we report a draft genome sequence of *P. variotii* no. 5; this draft was produced with the intent of investigating the mechanism of formaldehyde resistance. This is the first report of the genome sequence of any *Paecilomyces* species.

## GENOME ANNOUNCEMENT

*Paecilomyces* is widely distributed in soil, food products, and plant detritus. The genus *Paecilomyces* includes formaldehyde- and heat-resistant, opportunistic, nematophagous, and food spoilage fungi (1–5). *Paecilomyces variotii* is the anamorph of *Byssochlamys spectabilis* (6). *P. variotii* no. 5 (NBRC109023) was isolated in 2001 as a formaldehyde-resistant fungus from a soil sample taken at the Port of Nagasu, which is on the Ariake Sea in Kumamoto Prefecture, Japan. This strain degraded formaldehyde at concentrations as high as 2.0% within 20 days. To our knowledge, a genome sequence has not been published for any *Paecilomyces* species. Here, we report the draft genome sequence of *P. variotii* no. 5, which was generated with the intent of investigating the mechanism of this strain's formaldehyde resistance.

A whole-genome shotgun strategy was used to produce this draft sequence. The Genome Analyzer IIx (Illumina) was used to perform one shotgun run and 75-bp paired-end runs. This approach generated 1.59 Gbp (53.5-fold coverage) of genomic information and 21,200,000 sequencing reads. The Velvet version 1.0.03 software program (7) was used to assemble the sequencing data; 1,053 large contigs (≥100 bp) with N_50_ and N_90_ sizes of 137,200 bp and 30,647 bp, respectively, were produced. The maximum contig length is 475,531 bp. This draft genome of *P. variotii* no. 5 includes 29,762,401 bp (total sequence length) and a G+C content of 48.58%. The genome annotation of the obtained scaffolds was performed based on the Augustus version 2.7 software program (8), which was originally designed to predict genes in *Aspergillus oryzae* (9), and based on BLAST searches against a protein sequence database. These programs predicted 8,877 open reading frames, and each putative protein was assigned a predicted function. The average gene density is one gene per 1.617 kb, and on average, each gene has 3.45 exons. The average exon size is 468 bp. Approximately 21,774 introns, ranging from 34 to 4,394 bp, are present in the genome. The average intron size is 118 bp, and the average number of introns per open reading frame is 2.45.

Due to the ability of *P. variotii* no. 5 to degrade formaldehyde, BLASTp was used to screen the putative protein sequences for proteins related to formaldehyde metabolism (10). As a result, seven putative proteins of interest were found; these included the following formaldehyde degradation enzymes: two glutathione-dependent formaldehyde-activating enzymes (11), two *S*-(hydroxymethyl)glutathione dehydrogenases (12), two *S*-formylglutathione hydrolases (13), and a formate dehydrogenase (14). Interestingly, strain no. 5 also possesses putative genes encoding glutathione-independent formaldehyde dehydrogenases (15), which are enzymes that may catalyze biodegradation. Some of these putative formaldehyde-related genes were clustered immediately adjacent to one another on a single chromosome; they seemed to form a formaldehyde metabolism gene cluster.

### Nucleotide sequence accession numbers.

The whole-genome shotgun project has been deposited at DDBJ/EMBL/GenBank under the accession no. BAUL00000000. The version described in this paper is the first version, BAUL01000000.
